# Hybrid boron nitride-natural fiber composites for enhanced thermal conductivity

**DOI:** 10.1038/srep34726

**Published:** 2016-10-05

**Authors:** Changlei Xia, Andres C. Garcia, Sheldon Q. Shi, Ying Qiu, Nathaniel Warner, Yingji Wu, Liping Cai, Hussain R. Rizvi, Nandika A. D’Souza, Xu Nie

**Affiliations:** 1 Department of Mechanical and Energy Engineering, University of North Texas, Denton, TX 76203, USA; 2 Department of Materials Science and Engineering, University of North Texas, Denton, TX 76203, USA; 3 Department of Mechanical Engineering, Southern Methodist University, Dallas, TX 75205, USA.

## Abstract

Thermal conductivity was dramatically increased after adding natural fiber into hexagonal boron nitride (hBN)/epoxy composites. Although natural fiber does not show high-thermal conductivity itself, this study found that the synergy of natural fiber with hBN could significantly improve thermal conductivity, compared with that solely using hBN. A design of mixtures approach using constant fibers with increasing volume fractions of hBN was examined and compared. The thermal conductivity of the composite containing 43.6% hBN, 26.3% kenaf fiber and 30.1% epoxy reached 6.418 W m^−1^ K^−1^, which was 72.3% higher than that (3.600 W m^−1^ K^−1^) of the 69.0% hBN and 31.0% epoxy composite. Using the scanning electron microscope (SEM) and micro computed tomography (micro-CT), it was observed that the hBN powders were well distributed and ordered on the fiber surfaces enhancing the ceramic filler’s interconnection, which may be the reason for the increase in thermal conductivity. Additionally, the results from mechanical and dynamic mechanical tests showed that performances dramatically improved after adding kenaf fibers into the hBN/epoxy composite, potentially benefiting the composite’s use as an engineered material.

Environmental-friendly composites are increasingly needed and developed by utilizing natural fibers as reinforcements^1^. As one of the lignocellulosic fibers, kenaf (*Hibiscus cannabinus, L.* Malvaceae) fiber has been widely investigated due to its low density, low cost, high specific strength, and sustainability^2^,^3^,^4^,^5^,^6^,^7^,^8^. These composites have the potential to serve as the next-generation of materials^9^. The production of kenaf and related natural fibers are estimated the average yield of 288,000 t/year over 2007–2012, based on the report of Food and Agriculture Organization (FAO).

Fiberglass is one system used for electronic circuit boards. There is however an increased need for heat dissipation. The volume of microelectronic packaging is increasing annually and as technology transitions, increased disposal concerns and carbon footprint factors are being considered. The growth of under the hood automotive applications has led to increased demands for high thermal dissipation in the electronic devices used to deploy increased number of sensors. Therefore, heat dissipation of plays a significant role in modern electronic packaging applications^10^,^11^,^12^,^13^. Microelectronics temperatures have significant effect on the reliability of the electronic packaging materials, since the increasing temperature could dramatically decrease electronics life time and performance^14^,^15^,^16^,^17^. Therefore, it is important to optimize thermal conductivity and shape stability of electronic packaging materials for their wide applications^18^,^19^. High-thermal-conductivity composites are growing at exponential rates due to the desire advantages to be integrated on the next generation of light weight and high performance composites^20^,^21^.

High-thermal-conductivity materials are indispensable in the modern industrial^22^. Novel technologies were reported to manufacture high-thermal-conductivity materials, e.g. two-dimensional graphene nanoplatelets^23^, 3D boron nitride nanosheet^24^, network of graphite fillers^25^, nanosilver networks^26^, etc. However, these technologies are difficult to manufacture in large quantities. Polymeric composites have the potential to replace current electronic packaging, which need to provide high-thermal conductivity and high-electrical resistivity while possessing suitable mechanical properties. Many studies focused on the production of dielectric and thermally conductive polymer composites, using multiple polymers as matrix and fillers, such as, beryllium oxide, aluminum oxide, aluminum nitride and boron nitride^27^,^28^,^29^, however not only no significant increase in thermal conductivity was achieved, but also the high loadings of conductive ceramic caused a brittle composite^30^,^31^,^32^. Thus, to design and develop an electrically insulated and high-thermally conductive composite without compromising its mechanical properties is important.

In the recent studies, kenaf fiber showed excellent performance in hybrid composites with inorganic particles in the polymer matrix, such as calcium carbonate^33^,^34^, aluminum hydroxide^35^, iron oxide^36^, activated carbon^37^, etc. Hexagonal boron nitride (hBN) is a new filler that has gained increased interest for its dual contributions to high thermal conductivity and electrical insulative characteristics. Kenaf in and of itself is highly insulative. However recent results show increased thermal conductivity from combined thermally conductive and thermally insulative fillers when pathways for phonon conduction are created between the dispersed particulates. The main focus of this work is to investigate the thermal-conductivity enhancement provided by the addition of natural fibers to the hBN/epoxy composites. The structural analysis of the composites are used to explain the rationale of the enhancement. Moreover, mechanical and dynamic mechanical properties were examined.

## Results

**Figure 1:**
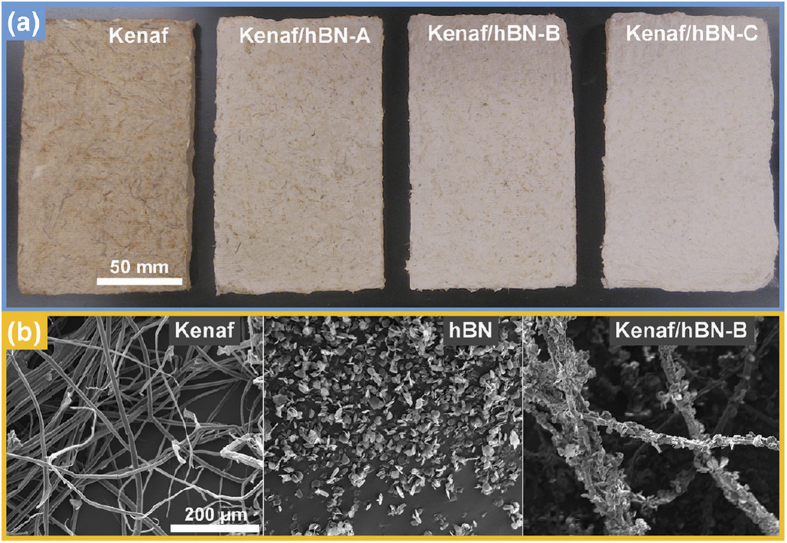
Photos of kenaf-fiber preforms (**a**), and SEM images of kenaf fiber, hBN powder, and hBN loaded kenaf fiber (**b**).

Different amounts of hBN, including 40, 60, and 80 g, were mixed with 23 g kenaf fibers (Table 1). The hBN loading efficiency was increased from 82.1% to 94.7%, when the feed of hBN was increased from 40 g to 80 g. The components of the hBN-loaded fibers are shown in Table 1. The photos of kenaf fibers and hBN-loaded kenaf fibers are shown in Fig. 1a. As clearly illustrated, the white hBN powders were absorbed onto the kenaf fibers, and the more the hBN loading onto kenaf fibers, the greater the white color of the preforms. Moreover, SEM was performed for the microtopography study (Fig. 1b). The SEM images of kenaf fibers, hBN powder and Kenaf/hBN-B are shown in Fig. 1b. From the SEM images of kenaf and hBN (Fig. 1b), it was measured that the average diameter of kenaf fiber was 11.5 ± 1.9 μm, and that of hBN was 13.8 ± 2.9 μm with a platelet shape. From the SEM image of Kenaf/hBN-B (Fig. 1b), it was shown that the hBN powders were absorbed onto the fiber surfaces, and the kenaf fiber worked as a foundation for those hBN powders.

The composites were fabricated using the hot-pressing technology. The densities and components of the composites and pure epoxy are presented in Table 2. It was found that the epoxy of the composites, including Kenaf/Epoxy/hBN-34.2%, Kenaf/Epoxy/hBN-43.6%, Kenaf/Epoxy/hBN-43.6%, and Epoxy/hBN-69.0%, were approximately constant in volume fraction (30.1–31.0%), and that of Kenaf/Epoxy composite was only slightly higher (34.0%). The density of the composites was increased with the higher hBN content, since the density (2.10 g cm^−3^) of hBN was higher than these of kenaf fiber (1.44 g cm^−3^) and epoxy (1.12 g cm^−3^).

### Thermal conductivity

**Figure 2:**
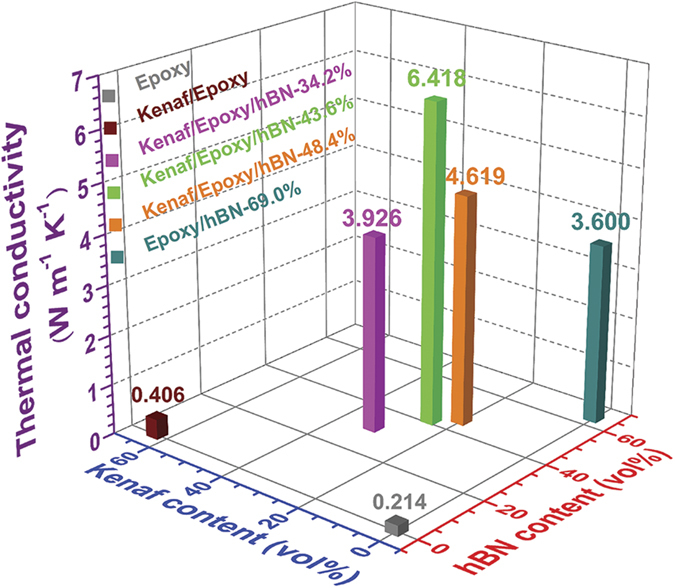
Thermal-conductivity results of the specimens.

Thermal conductivities of the composites and pure epoxy are shown in Fig. 2. Epoxy (0.214 ± 0.003 W m^-1^ K^−1^) and Kenaf/Epoxy (0.406 ± 0.001 W m^−1^ K^−1^) owned low thermal conductivities, and Keaf/Epoxy/hBN-34.2% (3.926 ± 0.064 W m^−1^ K^−1^), Kenaf/Epoxy/hBN-43.6% (6.418 ± 0.130 W m^−1^ K^−1^), Kenaf/Epoxy/hBN-48.4% (4.619 ± 0.212 W m^−1^ K^−1^), and Epoxy/hBN-69.0% (3.600 ± 0.349 W m^−1^ K^−1^) composites presented much higher thermal conductivities. As seen in Fig. 2, from right side to left side, the thermal conductivity of composite was increased with the increase in hBN content (or the decrease in kenaf content) at first. The thermal conductivity reached the highest value when an optimized ratio of Kenaf/hBN was achieved. After that, the thermal conductivity decreased. Surprisingly, the results showed that the thermal conductivity was dramatically improved after the addition of kenaf fibers into boron nitride/epoxy composite, though using kenaf fiber solely did not show the significant enhancement of thermal-conductivity. In the experiments, the highest thermal conductivity was Kenaf/Epoxy/hBN-43.6% composite (6.418 W m^−1^ K^−1^) containing 43.6% hBN. Compared with that of the Epoxy/hBN-69.0% composite (3.600 W m^−1^ K^−1^) containing 69.0% hBN, the hBN content of the Kenaf/Epoxy/hBN-43.6% composite was reduced by 36.8% but the thermal conductivity was improved by 72.3%. As shown in the SEM image of Kenaf/Epoxy/hBN-B (Fig. 1b), the hBN powders had a good arrangement on the kenaf fiber that serviced as a template. The good arrangement greatly helped hBN powders bind with each other tightly, which benefited the heat transfer through the composites.

### Structure study

**Figure 3:**
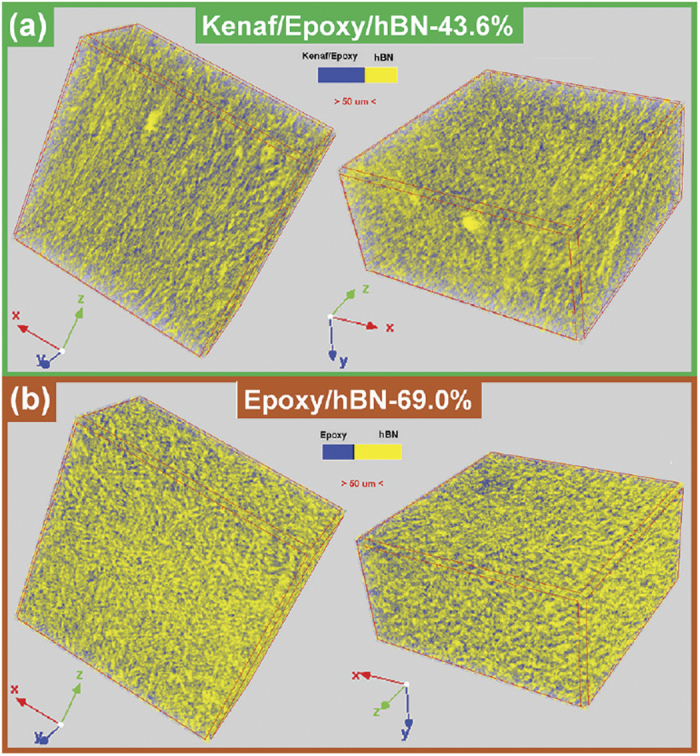
Mico-CT graphs of Kenaf/Epoxy/hBN-43.6% (**a**) and Epoxy/Hbn-69.0% (**b**) composites.

Micro-CT investigation of the Kenaf/Epoxy/hBN-43.6% (Fig. 3a) and Epoxy/hBN-69.0% composites (Fig. 3b) was performed for structural comparison. Compared to the Epoxy/hBN-69.0%, as seen in the 3D graphs (Fig. 3a,b), the hBN content (yellow phase) of the Kenaf/Epoxy/hBN-43.6% composite was much lower obviously, and the blue phase relatively larger. This blue phase in the 3D graphs of the Kenaf/Epoxy/hBN-43.6% composite (Fig. 3a) was probably contributed to the 30.1% resin and 26.3% kenaf fiber. The blue phase shows fiber-like shape in the 3D graphs of the Kenaf/Epoxy/hBN-43.6% composite (Fig. 3a), however, which presents uniform and random distribution of the Epoxy/hBN-69.0% composite (Fig. 3b). The hBN powders in the Kenaf/Epoxy/hBN-43.6% composite were more optimally arranged than those in the Epoxy/hBN-69.0% composite. This phenomenon was consistent with the SEM image of Kenaf/hBN-B fibers (Fig. 1b), in which the kenaf fiber could service as a template for improving the arrangement of hBN powders. From the results of thermal conductivities (measured in the Y-axis direction of the composite image in Fig. 3a,b), this arrangement increased the contacting opportunity among hBN powders.

### Mechanical properties

**Figure 4:**
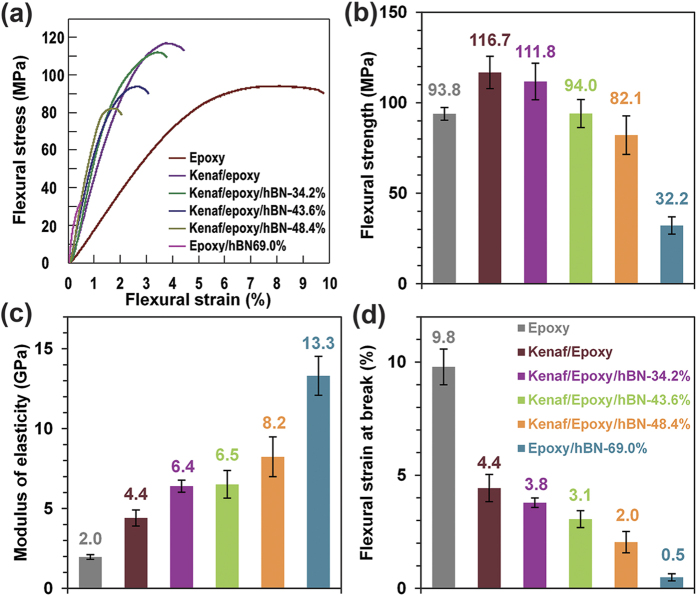
Flexural strain-stress curves (**a**), flexural strength (**b**), modulus of elasticity (**c**), and flexural strain at break (**d**) of the specimens.

The flexural strain-stress curves of the composites and pure epoxy are shown in Fig. 4a, and the results of flexural strength, modulus of elasticity, and flexural strain at break are presented in Fig. 4b–d, respectively. After adding kenaf fibers into epoxy (Kenaf/Epoxy), flexural strength and modulus of elasticity were increased from 93.8 MPa to 116.7 MPa, and from 2.0 GPa to 4.4 GPa, respectively, and flexural strain at break was decreased from 9.8% to 4.4%, presenting the relatively rigid characteristic of kenaf fiber rather than epoxy, which was consistent with the reported work^38^. With the increase of the hBN content, modulus of elasticity was increased from 4.4 GPa to 13.3 GPa, which is attributed to the rigid property of inorganic filler (hBN). However, flexural strength and flexural strain at break were decreased dramatically, from 116.7 MPa to 32.2 MPa, and from 4.4% to 0.5%, respectively. Compared to the composite without kenaf fiber (Epoxy/hBN-69.0%), flexural strength and flexural strain at break of the composite with highest thermal conductivity (Kenaf/Epoxy/hBN-43.6%) were improved by 192.0% and 527.0%, respectively. These improvements would greatly benefit the composites being used as engineering materials.

### Dynamic thermal mechanical properties

**Figure 5:**
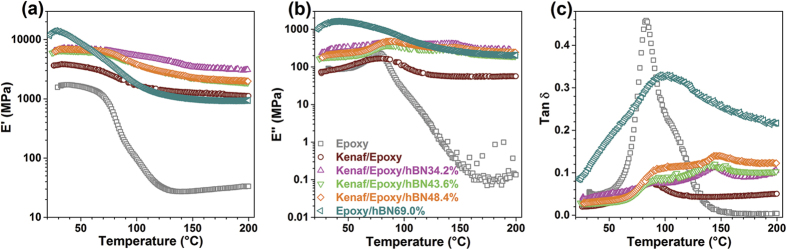
DMA results of the specimens, including storage modulus (**a**), loss modulus (**b**), and damping parameter (**c**).

DMA tests were performed and the results are shown in Fig. 5 and Table S1. E′ is an index of resilience that measures the energy storage capability of the material after elastic deformation. Figure 5a shows the E′ of the composites and pure epoxy. In general, E′ of the specimens decreased after around 27.8–38.9 °C as temperature increased because of the increasing chain mobility of the polymer matrix^39^. Kenaf/Epoxy/hBN composites showed larger E′ than the Kenaf/Epoxy composite and pure epoxy at overall the temperature range. For the Epoxy/hBN-69.0% composite, E′ dramatically jumped from the temperature of 27.8 °C to 100 °C, and showed slight lower E′ values than those of the Kenaf/Epoxy composite. The high temperature dependence of E′ would limit the application of the Epoxy/hBN-69.0% composite, especially applications as heat-transfer boards. However, after adding kenaf (Kenaf/Epoxy/hBN composites), this disadvantage could be greatly reduced. The E″ and tan δ curves for the composites and pure epoxy are shown in Fig. 5b,c, respectively. In the E″ curves of the specimens (Fig. 5b), the first peaks were shown at 76.4–87.5 °C, except for the Epoxy/hBN-69.0% composite at 40.4 °C, and the second peaks were shown only for Kenaf/Epoxy/hBN composites at 132–144 °C. In the tan δ curves (Fig. 5c), the first peaks were shown at 83.0–94.9 °C, and the second peaks were shown for only Kenaf/Epoxy/hBN composites at 142–149 °C. Additionally, the tan δ curves of the Epoxy and Epoxy/hBN-69.0% composite showed strong peaks, which indicated the glass transition temperatures were unambiguous. After adding kenaf fiber and hBN into epoxy simultaneously, the interaction between the components of the composites was improved^40^,^41^. It was indicated that the addition of kenaf fibers and hBN reduced the chain mobility of the epoxy matrix, so that the mechanical properties were enhanced at high temperatures.

## Discussion

The composite thermal conductivity was dramatically increased by incorporating kenaf fiber that does not have the ability to enhance thermal conductivity solely. The thermal conductivity of the 43.6%-hBN/26.3%-kenaf fiber/30.1%-epoxy composite was measured to be 6.418 W m^−1^ K^−1^, however, that of the 69.0%-hBN/31.0%-epoxy composite was only 3.600 W m^−1^ K^−1^. The increment of thermal conductivity was 72.3%, but the utilization of hBN was reduced by 36.8%. Using the SEM and micro-CT analysis, it was found that hBN was well distributed and ordered on the kenaf fiber surfaces, in other words, kenaf fiber serviced as a scaffold for hBN powders. This arrangement helped the hBN powders’ interconnection, resulting in a significant improvement in thermal conductivity. Additionally, the mechanical and dynamic mechanical analyses confirmed the property improvements of the composite after the addition of kenaf fibers.

## Methods

### Materials

The epoxy used in this experiment was Araldite LY 1556 US type epoxy from Huntsman Corp., USA, with Aradur 2964 (Huntsman Corp., USA) as the hardener. A mixing ratio of 2/1 (epoxy/hardener, vol/vol) was performed. The hBN powder (Material No: 2107825) was from Momentive Performance Material Inc., USA. Kenaf bast was obtained from Kengro Corp., USA, which was chopped into lengths of approximately 50.8 mm. Sodium hydroxide (NaOH) solution (5%, wt/vol) was prepared using NaOH beads (≥97%, Acros Organics) and deionized (DI) water obtained from a Millipore Milli-Q Integral Water Purification System.

### Kenaf/hBN preformation

First, 120 g kenaf bast (9.11% moisture content, measured by Mettler-Toledo HB43-S Moisture Analyzer) was added into a hermetical reactor (Parr Instrument Co. 251 M) with 1.8 L NaOH solution. Secondly, the alkali retting process was performed at 160 °C for one hour with approximate 100 rpm mechanical stirring. The saturated vapor pressure of the container was maintained at 0.60 MPa. After cooling to room temperature, the intensely ionic solution was removed from kenaf fibers first by gravity, then hand-squeezing, and subsequently running water, which was repeated three times. The achieved retted fiber yield was 38.5 ± 1.0%.

The hBN loaded fibers were prepared by mixing hBN powder and the retted fibers (after being washed, but not dried) into 1 L DI water, followed by being mechanically stirred for 1 h. The mixture was formed into a preform mat with a dimension of approximate 100 × 165 × 10 mm (width × length × thickness), and dried at 105 °C for 24 h. Different amounts of hBN were mixed with the same amount of kenaf fiber to prepare the samples. And the hBN loading efficiency was calculated considering the hBN loading and feeding amounts.

### Composites fabrication

A two-part cured epoxy system was used for the composite’s polymer matrix. Prior to the curing, the preformed mats were completely mixed with epoxy. The mixtures were then moved to a steel mold with a dia. of 40 mm. After being compressed by a hot press (Model # 3925, Carver Inc., USA) at 150 °C with a pressure of approximately 13.2 MPa maintained for 60 min, the specimens were then removed from the mold and kept for 7 h at room temperature before testing.

### Flexural properties tests

Three-point bending tests were carried out by the AGS-X universal testing machine (Shimadzu Corp., Japan) machine in accordance with the procedure described in ASTM D790 standard. Flexural strain-stress curves were obtained from the tests. The modulus of elasticity, flexural strength, and flexural strain at break of the composites were calculated by Eqs (1, 2, 3), respectively.


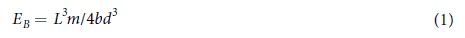



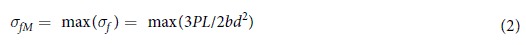



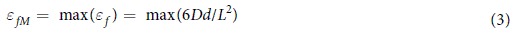


where *E*_*B*_ is modulus of elasticity; *L* is support span; *m* is slope of the tangent to the initial straight-line portion of the load-deflection curve; *b* is width of beam tested; *d* is depth; *σ*_*fM*_ is flexural strength; *σ*_*f*_ is flexural stress; *P* is load at a given point on the load-deflection curve; *ε*_*fM*_ is flexural strain at break; *ε*_*f*_ is flexural strain; D is maximum deflection of the center of the beam.

### Dynamic mechanical analysis

A Q800 dynamic mechanical analysis (DMA) tester (TA Instruments, USA) was employed. Specimens of 1 × 4 × 30 mm (thickness × width × length) were used for the DMA three-point bending tests with a gauge length of 25.4 mm. The temperature from 35 to 200 °C with a ramping speed of 5 °C min^−1^ was performed, and the frequency of the oscillation was set to be 1 Hz. The storage modulus (E′), loss modulus (E″) and mechanical loss factor (tan δ) were plotted as a function of temperature.

### Thermal conductivity tests

The samples used for thermal-conductivity tests measured approximately 40 mm in diameter and 5 mm in thickness. The thermal conductivities in the axial direction^42^,^43^ of the samples were taken using a TPS 1500 Thermal Constants Analyser (Hot Disk AB, Sweden). A Kapton sensor for isotropic/anisotropic probe measurements, operating at room temperature (20 °C), was placed over a sample (single sample testing) and enclosed inside the chamber.

### Microstructure analysis

A Quanta 200 environmental scanning electron microscope (SEM) with an accelerating voltage of 10 kV and a magnification of 250× was used to observe the kenaf fibers and hBN powders. Prior to the tests, the samples were coated by a gold sputtering coater for 1 min to prevent charging of the specimens by the SEM electron beam. Two composites were scanned with the X-ray micro computed tomography (micro-CT) SkyScan 1172 (Bruker Corp., USA) at a tube voltage and current of 40 kV and 250 μA, respectively. X-ray radiographs were taken with no filter, an angular step size of 0.08° and image pixel size of 1.99 μm. The collected radiographs were reconstructed with NRecon software, and then imported in Data Viewer and CTvox to obtain 3D images.

## Additional Information

**How to cite this article**: Xia, C. *et al*. Hybrid boron nitride-natural fiber composites for enhanced thermal conductivity. *Sci. Rep.* **6**, 34726; doi: 10.1038/srep34726 (2016).

## Figures and Tables

**Table 1:** Feedstocks for preparing the kenaf-fiber preforms and their components.

Specimen	Feed (g)		hBN loading efficiency^b^ (%)	Component (%)^c^	
	Fiber^a^	hBN		Fiber	hBN
Kenaf	23	—	—	100.0	—
Kenaf/hBN-A	23	40	82.1	50.5	49.5
Kenaf/hBN-B	23	60	92.7	37.6	62.4
Kenaf/hBN-C	23	80	94.7	30.7	69.3

^a^Alkali-retted kenaf fiber.

^b^hBN loading efficiency = (amount of hBN loaded onto kenaf fiber)/(amount of hBN feed).

^c^In volume fraction.

**Table 2:** Densities and components of pure epoxy and the composites.

Specimen	Density (g cm^−3^)	Component (%)^c^		
		Resin^a^	Fiber^b^	hBN
Epoxy	1.12	100.0	—	—
Kenaf/Epoxy	1.29	34.0	66.0	—
Kenaf/Epoxy/hBN-34.2%	1.52	30.9	34.9	34.2
Kenaf/Epoxy/hBN-43.6%	1.65	30.1	26.3	43.6
Kenaf/Epoxy/hBN-48.4%	1.66	30.2	21.4	48.4
Epoxy/hBN-69.0%	1.76	31.0	—	69.0

^a^Epoxy/hardener (2/1, vol/vol).

^b^Alkali-retted kenaf fiber.

^c^In volume fraction.
